# Supplementary material for: Causal Relationship between Aging and Anorexia Nervosa: A White-Matter-Microstructure-Mediated Mendelian Randomization Analysis
Source: Biomedicines. 2024 Aug 16;12(8):1874. doi: 10.3390/biomedicines12081874 (PMC11351342; doi:10.3390/biomedicines12081874)
Supplement: Supplementary file 1 [file biomedicines-12-01874-s001.zip › Supplementary Figure/Figure S8/Leaveoneout plot of MR analysis for TL to ubm-b-2075.pdf]

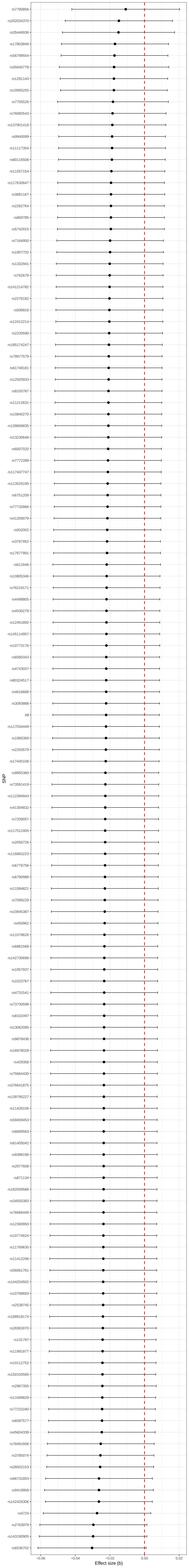
rs7790856
rs202034370
rs35446936
rs17803849
rs56799554
rs35640778
rs1291143
rs10905255
rs7705526
rs76065543
rs137901416
rs9940099
rs11117354
rs80116508
rs11557154
rs117630647
rs3891167
rs2282764
rs869785
rs5742915
rs7164950
rs1907702
rs1332941
rs762679
rs141214782
rs2276182
rs939916
rs12412214
rs2230590
rs185174247
rs79977579
rs61748181
rs12925933
rs8105767
rs11212631
rs10840270
rs139669835
rs13230646
rs6007020
rs7772289
rs117407747
rs113525195
rs6751209
rs77732866
rs41269079
rs932002
rs3767952
rs17677991
rs611646
rs10805346
rs76219171
rs4498805
rs4530278
rs12451892
rs145114957
rs10773176
rs6590343
rs4743037
rs80324517
rs4616688
rs3093888
All
rs117034449
rs1985369
rs2293579
rs17445108
rs9955360
rs73581419
rs112394943
rs41304832
rs7209057
rs117512405
rs2056726
rs116863223
rs6776756
rs6790988
rs11584821
rs7099229
rs10845387
rs450962
rs11579626
rs6881568
rs142730696
rs1957937
rs1023767
rs4731541
rs73730598
rs8102497
rs13062095
rs9878436
rs16978028
rs429358
rs75664430
rs376641875
rs139795227
rs11426156
rs59409453
rs6669563
rs61405042
rs9398196
rs2977608
rs871134
rs182059586
rs34550383
rs76666449
rs12369950
rs10774624
rs11769630
rs11412296
rs56061761
rs144204502
rs10768683
rs2538745
rs188918174
rs28363070
rs131797
rs11991877
rs10112752
rs150150565
rs2967355
rs11699829
rs77231040
rs6587577
rs45604339
rs78491606
rs3785074
rs28502153
rs66731853
rs9419958
rs142426306
rs4724
rs2763979
rs143190905
rs6536702
SNP
−0.06
−0.04
−0.02
0.00
0.02
Effect size (b)
